# Clinical results of definitive radiotherapy for local recurrent kimura disease in the head and neck after surgery: A retrospective study

**DOI:** 10.1002/pro6.1238

**Published:** 2024-08-21

**Authors:** Wenlong Lv, Shan Li, Feng Liu, Wangui Xue, Feibao Guo, Jinsheng Hong

**Affiliations:** ^1^ Department of Radiotherapy Cancer Center The First Affiliated Hospital of Fujian Medical University Fuzhou China; ^2^ Department of Radiotherapy National Regional Medical Center Binhai Campus of the First Affiliated Hospital Fujian Medical University Fuzhou China; ^3^ Key Laboratory of Radiation Biology of Fujian higher education institutions The First Affiliated Hospital Fujian Medical University Fuzhou China

**Keywords:** Kimura disease, radiotherapy, head and neck, tumor recurrence

## Abstract

**Objective:**

To evaluate the efficacy and safety of definitive radiotherapy in selected patients with local recurrence of Kimura disease of the head and neck after surgery.

**Methods:**

This retrospective study collected the clinical data of 14 patients with postoperative recurrence of Kimura disease of the head and neck who received definitive radiotherapy at the First Affiliated Hospital of Fujian Medical University between 2006 and 2022. The radiation dose ranged from 28 to 40 Gy. Its efficacy and safety were analyzed.

**Results:**

During follow‐up, ranging from 17 to 168 months, local control was achieved in 13 (92.9%) of the 14 patients with postoperative recurrence. There were no serious late toxicities except for mild xerostomia in four (28.6%) patients; the patients’ peripheral blood eosinophil count dropped from 1.73×10^9^/L before treatment to 0.42×10^9^/L after treatment, and the eosinophil percentage dropped from 20.64% to 9.78%. Both changes were statistically significant (*p*<0.001).

**Conclusions:**

The findings of the study suggest that definitive radiotherapy is a viable and effective alternative to repeated surgery for managing recurrent Kimura disease of the head and neck, with significant response rates and a good safety profile. Peripheral blood eosinophil counts and percentages serve as simple and reliable biomarkers for monitoring Kimura disease progression and treatment responses.

## Graphical abstract

The graphic on the left represents definitive radiotherapy for selected patients with local recurrence of kimura disease in the head and neck after surgery.

The figure on the right is divided into upper and lower parts. The upper part

The graph is divided into upper and lower parts. The upper part

‐ Use a shield (for safety) and a thumbs‐up icon (for efficacy).

‐ Text below the icon: “Radiation therapy is effective and safe for recurrent Kimura disease.”

‐ The lower half includes blood drop icons representing biomarkers.

‐ Add text: “Peripheral blood eosinophil count and percentage are reliable biomarkers.”

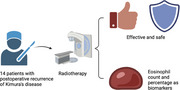

## 1 INTRODUCTION

Kimura disease (KD) is a rare, chronic proliferative inflammatory disease of the lymphoid tissue.^1^ It was first described by Chinese scientists as an “eosinophilic hyperplastic lymphogranuloma” in 1937,^2^ but it was not until 1948 when Kimura et al. in Japan published definitive histological characteristics that it became known as KD.^3^ KD occurs primarily in the head and neck region, particularly in the parotid and retroauricular areas of the scalp.^4^ The most commonly used local treatments are surgical excision, radiation therapy, and a combination of surgery and radiation therapy. ^5^ Although most patients initially undergo local surgical excision, clinical observations have shown that some experience rapid postoperative recurrence.^6^ To our knowledge, the treatment of locally recurrent KD after surgical failure remains challenging, with no consistent guidelines or widely accepted recommendations. Given the head and neck anatomical constraints, desire to preserve function and aesthetics, and patients' reluctance to undergo multiple surgeries, the use of surgery is often limited in most cases. Radiation therapy continues to be used as an adjunctive treatment for KD of the head and neck, with related research being scarce and mostly found in case reports and systematic reviews.^7^ Therefore, in this study, we aimed to explore the effectiveness and safety of radiotherapy as a treatment for locally recurrent KD after surgery and provide recommendations for the treatment of locally recurrent KD of the head and neck.

## 2 PATIENTS AND METHODS

### 2.1 Patient selection

The case data of 14 patients with postoperative recurrence of KD who underwent radical radiotherapy in the Department of Radiotherapy of the First Affiliated Hospital of Fujian Medical University between January 2006 and December 2022 were retrospectively analyzed. All patients had lesions in the head and neck, had undergone curative‐intent surgery that was at least macroscopically complete, and subsequently developed pathologically diagnosed with local recurrent KD. The patients’ clinical data, laboratory examinations, imaging, pathological examinations, treatment history, outcomes, and other clinical data were summarized and analyzed.

### 2.2 Treatments

Fourteen patients underwent 15 radiotherapy rounds. Intensity‐modulated radiation therapy (IMRT) or volumetric modulated arc therapy  (VMAT) was used in the radiotherapy plan. The gross tumor volume (GTV) was the visible tumor lesion or postoperative tumor bed in images (positioning computed tomography or magnetic resonance imaging), and the clinical target volume (CTV) was the GTV extension and included relevant lymphatic drainage areas. The planning target volume (PTV) was created by creating a 3–5 mm geometric expansion from the CTV and delineating organs at risk. The median dose of 95% PTV was 36 Gy; the dose range was: 28–40 Gy, 14–20 fractions, and the fractionated dose was 2.0‐2.12 Gy, 1 fraction per day, 5 fractions per week.

### 2.3 Following up

The patients were followed‐up until March 2024. The follow‐up duration ranged from 17 to 168 months, with a median follow‐up of 86 months. Follow‐up methods included outpatient visits and telephone follow‐ups. Follow‐up content included clinical symptoms and laboratory and imaging examinations. Recurrence was confirmed histopathologically. Acute and late toxicities were scored according to the Common Terminology Criteria for Adverse Events v5.0.

### 2.4 Statistical analysis

All data were analyzed using the SPSS version 23.0 software (IBM, NY, USA). Counts and percentages were used to describe categorical variables. Quantitative variables were described using mean and standard deviation. A paired *t*‐test was used to compare differences between groups. Statistical significance was set at *p*<0.05.

## 3 RESULTS

### 3.1 Clinical Characteristics

There were 14 patients with recurrent KD, including 12 male and 2 female patients. The age of onset ranged from 19 to 75 years, with a median of 41 years. All patients underwent one or more curative‐intent surgeries before being referred to the Department of Radiotherapy, with six patients undergoing two surgeries and eight patients undergoing one surgery. The median interval from surgical excision to recurrence was 36 months, ranging from 4 to 156 months. Recurrence occurred at the original site and surroundings. Thirteen patients presented with painless subcutaneous masses or regional lymphadenopathy in the head and neck area as their chief complaint, and one patient presented with a nasal mass. The main physical examination findings were irregular masses, firm and fixed, with poor mobility, accompanied by skin and subcutaneous itching (35.7%). The parotid gland was the most common site (71.4%), followed by the ear and neck. Unilateral involvement was more common (71.4 %) than bilateral involvement was (28.6%). Five patients (35.7%) presented with a single isolated lesion, whereas nine (64.3%) presented with multiple lesions. The median (maximum) diameter of the masses was 4.0 cm, ranging from 2.0 to 8.0 cm. A full‐body examination did not reveal involvement of the extracranial organs or lymph nodes in the head and neck area. The patient characteristics are presented in Table 1, and their details are summarized in Table 2.

**TABLE 1:** Characteristics of the 14 patients with Kimura disease.

Characteristic	No. of patients(%)
Sex	
Male	12 (86.7)
Female	2 (14.3)
Age at onset (years)	
≥40	8 (57.1)
<40	6 (42.9)
Lesion number	
Single	5 (35.7)
Multiple	9 (64.3)
Tumor diameter (cm)	
≥4	10 (71.4)
<4	4 (28.6)
Parotid gland involvement	
Yes	10 (71.4)
No	4 (28.6)
Pruritus	
Yes	5 (35.7)
No	9 (64.3)
Peripheral blood eosinophil	
Increase	13 (93.3)
Normal	1 (7.1)
Peripheral blood eosinophil percentage	
Increase	14 (92.9)
Interval to recurrence after first excision(m)	
>36	5 (35.7)
≤36	9 (64.3)
Number of surgeries	
1	8 (57.1)
2	6 (42.9)

**TABLE 2:** Case summary of local recurrent Kimura disease treated with radiation therapy.

KD	Sex/age (years)	Initial location	Single/multiple(1/2)	Unilateral/bilateral(1/2)	Resections(n)	First postoperative recurrence time (m)	Secondary treatment	Second recurrence time (m)	Third treatment	Follow‐up (m)/ status
01	F/59	Parotid gland/LN	2	1	1	4	RT	NR	NR	74/NED
02	M/34	Parotid gland/ postauricular	2	2	2	60	Surgery	36	RT	132/NED
03	M/75	Temporal/Eye socket	2	1	1	36	RT	NR	NR	144/Dead
04	M/21	Cheek/LN	2	1	1	6	RT	NR	NR	17/NED
05	M/39	Parotid gland	1	1	2	60	Surgery	12	RT	130/NED
06	M/19	Parotid gland/ postauricular	2	1	2	12	Surgery	24	RT	Lost tofollow‐up
07	M/19	Parotid gland/LN	2	2	1	12	RT	NR	NR	168/NED
08	F/51	Postauricular/LN	2	1	2	72	Surgery	144	RT	24/NED
09	M/50	Parotid gland	1	1	1	48	RT	NR	NR	86/NED
10	M/62	Parotid gland	1	1	1	156	RT	NR	NR	96/Dead
11	M/45	Nasal cavity	1	1	1	36	RT	108	RT	166/NED
12	M/63	Parotid gland/LN	2	2	2	24	Surgery	6	RT	72/NED
13	M/34	Parotid gland	2	2	2	36	Surgery	60	RT	144/NED
14	M/41	Parotid gland	1	1	1	36	RT	NR	NR	62/NED

Abbreviations: F, female; LN, lymph node; M, male; NED, no evidence of disease; NR, no reply; RT, radiotherapy.

### 3.2 Histopathological findings

Among the 14 patients, 1 was diagnosed by core needle biopsy and 13 were diagnosed with KD by biopsy surgery. The most common histological features were tissue eosinophilia (100%) and lymphoid follicular hyperplasia (92.9%). Proliferative vascular lesions and eosinophilic microabscesses were observed in some cases.

### 3.3 Laboratory test results

Peripheral blood cell analysis of the patients before radiotherapy showed that 13 (92.8%) had elevated eosinophil counts. Peripheral blood eosinophil percentages were elevated in all the patients.

Before radiotherapy, the mean absolute eosinophil count was (1.73±0.95)×10^9^/L, which significantly decreased to (0.42±0.21)×10^9^/L after treatment. The paired *t*‐test yielded a *t*‐value of 4.83, indicating a significant reduction (*p*<0.001). Similarly, the mean percentage of eosinophils significantly decreased from (20.64±8.31)% before radiotherapy to (9.78±4.43)% after it, *t* = 5.03, *p*<0.001.

### 3.4 Prognosis

All 14 patients with postoperative recurrence achieved complete remission after radiotherapy. Most lesions regressed rapidly during radiation treatment. The associated itching resolved after radiotherapy. One patient in Singapore experienced recurrence within the radiation field 108 months after radiotherapy, with no recurrence observed during follow‐up after receiving a second round of radiotherapy. Two patients who underwent radiotherapy for recurrent KD died. The first patient died 144 months after undergoing radiotherapy, at the age of 87 years; the cause of death was cerebral infarction. The second patient died 96 months after radiotherapy, at the age of 71 years; the cause of death was heart failure. Similar to the first patient, there were no records of disease recurrence during the time between the radiotherapy and death for the second patient. The remaining patients showed no evidence of the disease at their last follow‐up visit. No concurrent systemic diseases were observed.

### 3.5 Toxicity

During radiotherapy, patients were monitored weekly for acute adverse reactions. All patients tolerated the treatment well and no patient experienced an interruption due to adverse reactions. No adverse reactions greater than grade 3 were reported during the acute phase. Among the 14 patients who received radiotherapy in our hospital, 4 (28.6%) developed grade 2 oral mucositis, 5 (35.8%) developed grade 1 oral mucositis, 1 (7.1%) developed grade 2 radiation dermatitis, and grade 1 radiation dermatitis occurred in 50.0% of patients; no other hematological or gastrointestinal adverse reactions were observed. After radiotherapy, four patients (28.6%) experienced long‐term dry mouth symptoms. One patient (7.1%) had grade 2 xerostomia and three (21.4%) had grade 1 xerostomia; no obvious long‐term adverse reactions were observed in the remaining patients.

### 3.6 Case description

**FIGURE 1:**
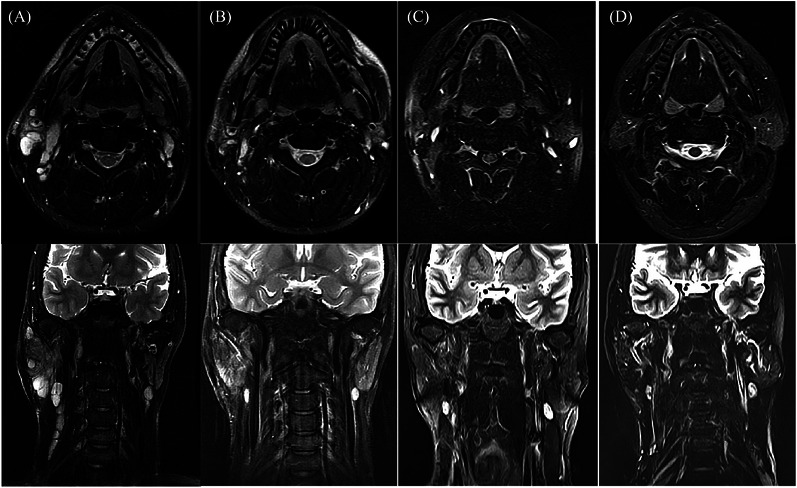
T2 magnetic resonance images of patient KD 07 (19 year‐old man). (A) Before, (B)1 month after, (C) 2 years after, and (D) 14 years after radiotherapy.

**FIGURE 2:**
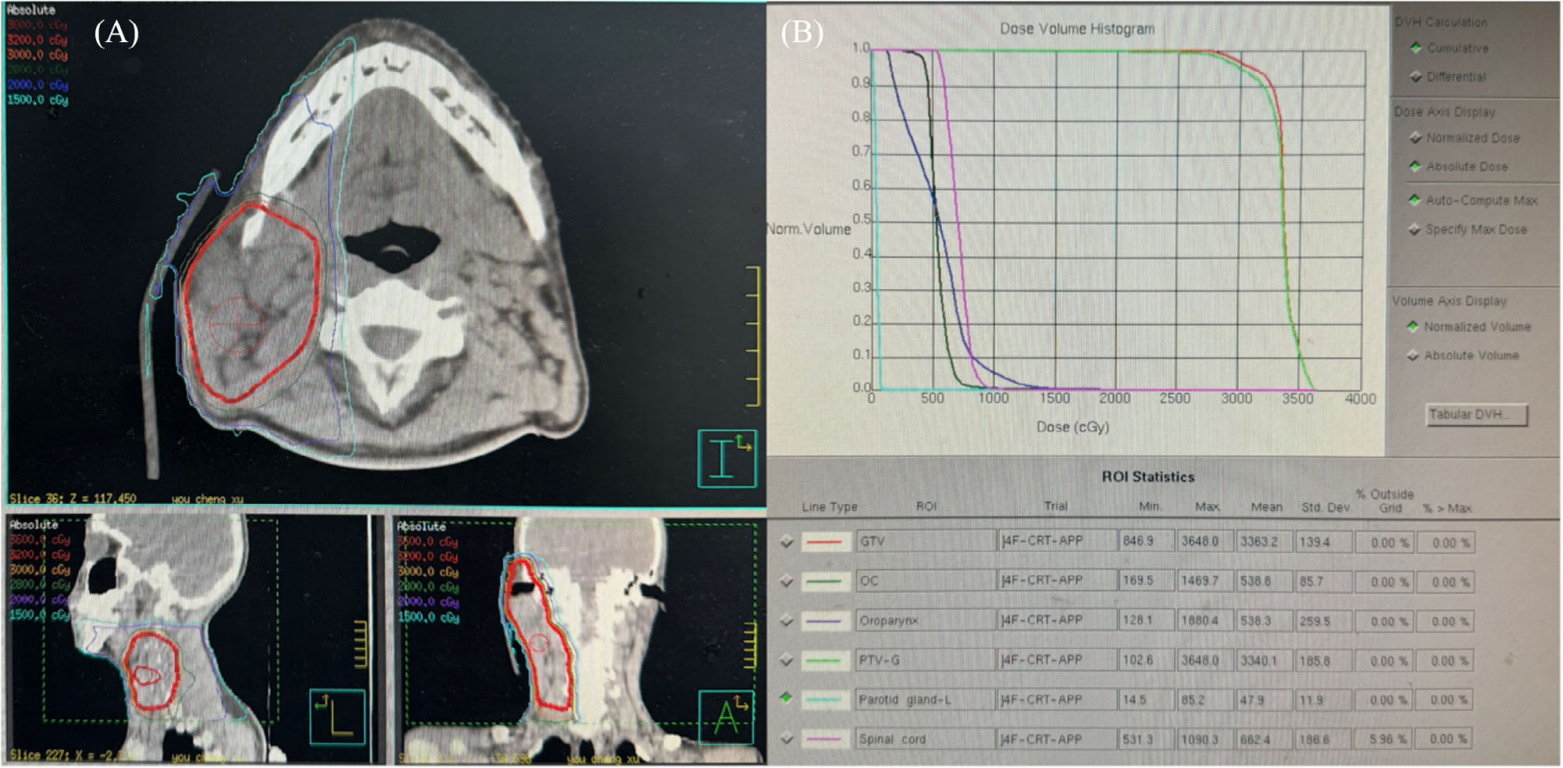
(A) Dose distribution for gross tumor volume. (B) Dose volume histogram for the planned target volume and organs at risk.

Patient KD07 was a 19‐year‐old man who was diagnosed with KD and underwent surgery in 2004. Recurrence occurred 1 year after surgery, and the parotid gland and neck masses that had been present gradually enlarged over 5 years. He was referred to the radiotherapy department for evaluation and treated with radiotherapy. Radiation therapy uses 6‐Mv photons to cover the visible mass region, with a GTV to PTV margin of 3 mm. A total dose of 32 Gy was prescribed in one course of 2 Gy/5 fractions/1 week. The patient recovered well after radiotherapy and has been followed‐up for 14 years without any signs of residual mass or recurrence. Figure 1 records the T2 magnetic resonance images of patient KD07 before and after radiotherapy (1 month, 2 years, and 14 years after radiotherapy). Figure 2 shows the dose distribution for the GTV and the dose‐volume histogram for the PTV and organs at risk.

## 4 DISCUSSION

The findings of this retrospective study provide essential insights into the clinical efficacy and safety of definitive radiotherapy for the treatment of locally recurrent KD of the head and neck following surgical intervention. Despite the rarity of KD, which is characterized by its chronic proliferative and inflammatory nature, post‐surgery local recurrence poses a significant treatment challenge, as highlighted by our findings and previous studies.^8^ In particular, among patients with KD of the head and neck, the reluctance towards multiple surgeries due to anatomical, functional, and aesthetic considerations underscores the need for alternative, effective treatment modalities. Our study, encompassing a cohort of 14 patients who experienced postoperative recurrence and subsequently underwent radiotherapy, aimed to elucidate the therapeutic potential and limitations of radiotherapy in managing this challenging clinical scenario.

KD usually progresses inertly, and patients often seek medical treatment after symptoms or signs affect their daily lives.^9^ The clinical manifestations are usually painless subcutaneous tumors or enlarged lymph nodes in the head and neck, with the parotid gland being the most commonly affected. In previous reports, surgery was considered the most useful treatment.^10^ However, postoperative recurrence is common. In our study, the median interval from surgical excision to recurrence was 36 months, with lesions typically recurring at or near the original site, underscoring the persistent nature of KD. This distribution is consistent with the known predilection of KD for lymphoid tissues in these anatomical areas.

Because KD is a benign tumor, local radiotherapy has always been used as an adjuvant therapy in its treatment.^11^, ^12^ The application of IMRT or VMAT technology in administering radiotherapy, as observed in our study, showcases the advancement in radiotherapeutic techniques, allowing for precise targeting and sparing of normal tissues, which is crucial in treating diseases with a predilection for anatomically complex regions, such as KD in the head and neck. Radiotherapy was highly effective in achieving complete remission in all patients, with significant reductions in the absolute eosinophil counts and percentages. Symptoms of skin itching disappeared after radiotherapy. These findings are particularly noteworthy, as they suggest that radiotherapy not only controls the local tumor but also significantly affects the inflammatory component of KD. The median eosinophil count significantly decreased from 1.73×10^9^/L to 0.42×10^9^/L, and eosinophil percentages significantly decreased from 20.64% to 9.78% after radiotherapy. These results highlight the potential of peripheral blood eosinophil counts and percentages as simple and reliable biomarkers for monitoring disease progression and treatment responses in patients with KD.

Owing to the long‐term survival of patients with KD, the safety of radiotherapy was another focal point of our analysis. To date, there have been almost no reports on the toxicity and side effects of KD radiotherapy, and only one document has described mild dry mouth symptoms after radiotherapy.^6^ In our study, the treatment was well‐tolerated by all participants. Only four (28.6%) patients experienced long‐term dry mouth symptoms after radiotherapy; 75% of them only had grade 1 xerostomia, and the remaining patients had no obvious long‐term adverse reactions. This is crucial considering the proximity of KD lesions to critical structures in the head and neck, necessitating precise radiation delivery to minimize harm while maximizing therapeutic effects. Despite the overall efficacy, one patient experienced recurrence inside the initial radiation field at 108 months, indicating the need for vigilant long‐term monitoring. However, the patient achieved CR after a second round of radiotherapy in Singapore. Additionally, the deaths of two patients from causes unrelated to KD highlight the importance of comprehensive management strategies that address not only KD but also other potential comorbid conditions.

## 5 CONCLUSION

This study underscores the potential of definitive radiotherapy as a viable and effective treatment alternative for patients with locally recurrent KD of the head and neck, thereby reducing the need for repeated surgeries. Radiotherapy has emerged as the cornerstone treatment modality for this patient population because it provides substantial remission rates and demonstrates a favorable safety profile. Peripheral blood eosinophil counts and percentages are simple and reliable biomarkers for monitoring disease progression and treatment response in patients with KD. Future research should focus on expanding these findings through prospective studies and exploring the integration of radiotherapy with multidisciplinary treatments.

## CONFLICT OF INTEREST STATEMENT

The authors declare no conflict of interest.

6

## ETHICS STATEMENT

This study was approved by the Institutional Review Board of the First Affiliated Hospital of Fujian Medical University（MTCA, ECFAH of FMU[2015]084‐2).

## Data Availability

The data that support the findings of this study are available from the corresponding author upon reasonable request.
